# Season and myocardial injury in patients with ST-segment elevation myocardial infarction: A cardiac magnetic resonance imaging study

**DOI:** 10.1371/journal.pone.0211807

**Published:** 2019-02-08

**Authors:** Ik Hyun Park, Woo Jin Jang, Hyun Kyu Cho, Ju Hyeon Oh, Woo Jung Chun, Yong Hwan Park, Mirae Lee, Young Bin Song, Joo-Yong Hahn, Seung-Hyuk Choi, Sang-Chol Lee, Hyeon-Cheol Gwon, Yeon Hyeon Choe

**Affiliations:** 1 Division of Cardiology, Department of Internal Medicine, Samsung Changwon Hospital, Sungkyunkwan University School of Medicine, Changwon, Republic of Korea; 2 Division of Cardiology, Department of Medicine, Heart Vascular Stroke Institute, Samsung Medical Center, Sungkyunkwan University School of Medicine, Seoul, Republic of Korea; 3 Department of Radiology, Cardiovascular Imaging Center, Samsung Medical Center, Sungkyunkwan University School of Medicine, Seoul, Republic of Korea; University of Bologna, ITALY

## Abstract

**Background:**

Little is known about the causality and pathological mechanism underlying the association of seasonal variation with myocardial injury in patients with ST-segment elevation myocardial infarction (STEMI).

**Objective:**

We evaluated the association of seasonal effect with myocardial injury using cardiovascular magnetic resonance (CMR) imaging in STEMI patients undergoing primary percutaneous coronary intervention (PCI).

**Methods:**

In 279 patients undergoing primary PCI for STEMI, CMR was performed for a median of 3.3 days after the index procedure. Of these, STEMI occurred in 56 patients in the winter (Winter group), 80 patients in the spring (Spring group), 76 patients in the summer (Summer group), and 67 patients in the autumn (Autumn group), respectively. We compared myocardial infarct size, extent of area at risk (AAR), myocardial salvage index (MSI) and microvascular obstruction (MVO) area as assessed by CMR according to the season in which STEMI occurred.

**Results:**

In the CMR analysis, the myocardial infarct size was not significantly different among the Winter group (21.0 ± 10.5%), the Spring group (19.6 ± 11.5%), the Summer group (18.6 ± 10.6%), and the Autumn group (21.1 ± 11.3%) (*P* = 0.475). The extent of AAR, MSI, and MVO areas were similar among the four groups. In the subgroup analysis, myocardial infarct size, extent of AAR, MSI, and MVO were not significantly different between the Harsh climate (winter + summer) and the Mild climate (spring + autumn) groups.

**Conclusions:**

Seasonal influences may not affect advanced myocardial injury in STEMI patients undergoing primary PCI.

## Introduction

Seasonal variations influence the incidence of acute myocardial infarction (MI) [1]. Previous studies have reported that acute MI occurs more frequently in cold and hot weather, and that ambient temperature may play an important role in the development of acute MI [2]. Evidence in support of this data considers various mechanisms, such as a potential effect of temperature on platelet activation, blood viscosity, and vascular resistance [2–4]. Kloner et al. [5] investigated seasonal variations in myocardial perfusion using enzymatic infarct size as estimated by the cumulative release of cardiac enzymes and reported that smaller infarct size was observed in the summer, but the causality and pathological mechanisms underlying the association of seasonal effects with myocardial injury remained unclear. Cardiovascular magnetic resonance (CMR) imaging can precisely assess the extent of myocardial injury and salvaged myocardium in acute MI patients [6,7]. We evaluated the association between seasonal variation and myocardial injury as assessed by CMR imaging in STEMI patients undergoing primary PCI.

## Methods

This study was approved by the institutional review board of Samsung Medical Center and Samsung Changwon Hospital, Sungkyunkwan University School of Medicine, respectively and all subjects provided written informed consent to participate in this study.

### Study population

**Fig 1:**
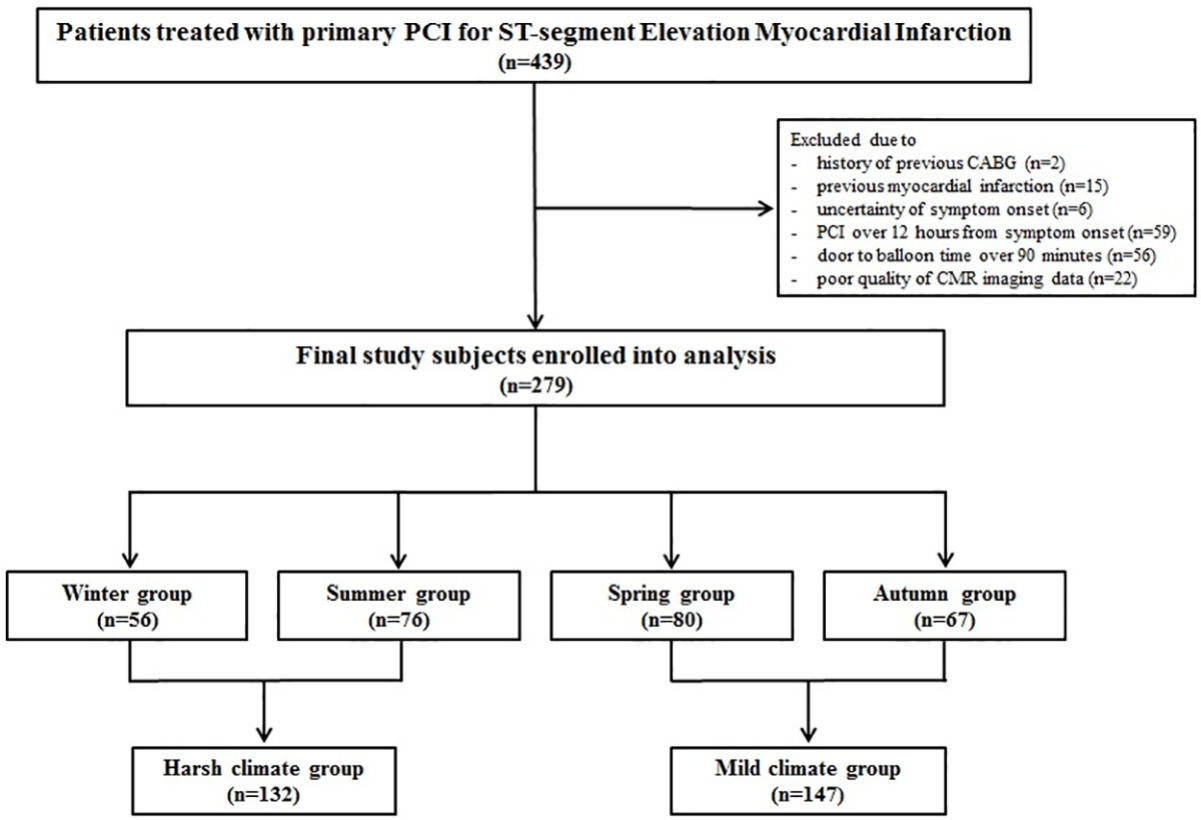
Schematic of study cohort selection. CABG = coronary artery bypass grafting; CMR = cardiac magnetic resonance; PCI = percutaneous coronary infarction.

Between December 2007 and July 2016, 439 consecutive patients were eligible for enrollment in this study after presenting with STEMI and undergoing CMR at the Samsung Medical Center, Seoul, Republic of Korea and the Samsung Changwon Hospital, Gyeongsangnam-do, Republic of Korea. The inclusion criteria for this study were: 1) patients treated with primary PCI within 12 hours after symptom onset, and 2) patients that underwent CMR after the index procedure. The exclusion criteria were: 1) previous coronary artery bypass grafting, 2) history of previous MI, 3) patients received reperfusion therapy over 12 hours from symptom onset, 4) insufficient information regarding symptom onset time, 5) door to balloon time over 90 minutes, and 6) patients with poor-quality CMR imaging data for analysis. Finally, 279 patients were included in this study (Fig 1).

### Season and definition of study group

South Korea lies in the temperate zone and has four distinct seasons, with the annual mean temperature ranging from 10 to 16°C and the climate extreme ranging from -32.6 to 40.0°C according to Meteorological Administration climate [8]. The four seasons are winter (December to February), spring (March to May), summer (June to August) and autumn (September to November). The winter months are cold and dry as a result of continental high-pressure systems, while summer months have high temperatures and humidity due to the North Pacific high-pressure system [2]. The spring and autumn months generally are mild in comparison as a result of the impact of migratory anticyclones [2]. In the present study, patients were divided into four groups according to when their STEMI occurred (i.e., Winter, Spring, Summer and Autumn).

### Study outcomes

The primary outcome was myocardial infarct size (% of left ventricle or -cular [LV]) as assessed by CMR imaging according to the occurrence season of acute MI. The secondary outcomes included extent of the area at risk (AAR; % of LV), myocardial salvage index (MSI), and microvascular obstruction (MVO) area (% of LV).

### Data collection and definition

Baseline characteristics, angiographic findings, and CMR data were prospectively recorded in the dedicated registry. Blood samples for N-terminal pro B-type natriuretic peptides and creatine kinase myocardial bands (CK-MB) were drawn from patients before primary PCI. Serum CK-MB levels were measured every eight hours from the index procedure until peak values were confirmed. The baseline left ventricular ejection fraction (LVEF; %) was measured by transthoracic echocardiography using the Simpson’s method immediately after primary PCI [9]. The Killip classification was determined upon patient arrival or before primary PCI [10]. STEMI was defined as an ST-segment elevation of more than 1 mm in two or more contiguous leads or a presumably new-onset left bundle branch block on electrocardiogram. Multi-vessel disease was defined as stenosis >50% in more than two coronary arteries. The thrombolysis in myocardial infarction (TIMI) flow grade and myocardial blush grade (MBG) were evaluated using the final angiogram, as defined previously [11]. All baseline and procedural cine coronary angiograms were reviewed and quantitatively analyzed at the angiographic core laboratory of our institution.

### CMR imaging analysis

All measurements were made in the Samsung Medical Center-CMR core laboratory using validated software (ARGUS; Siemens Medical Systems, Erlangen, Germany). Infarct size and extent of MVO were assessed on delayed enhanced images, whereas the AAR was measured on T2-weighted images. Two experienced radiologists blinded to patient information performed measurements based on visual assessment. After acquiring short-axis images at the end-diastole and end-systole, the endocardial borders were traced. The Simpson algorithm was then used to calculate the LV end-diastolic volume (LVEDV), the LV end-systolic volume (LVESV), and the LVEF. Infarct size was calculated from summation of the area with delayed hyperenhancement within each segment of the short-axis images. This value was multiplied by slice thickness to cover the entire left ventricle. Endocardial and epicardial borders were planimetered to calculate the myocardial area. They were then summed to calculate LV myocardial volume using the same method. Infarct size was expressed as the percentage of affected LV myocardial volume. T2-weighted images were used to determine the presence of hemorrhagic infarction [12]. AAR was quantified on T2-weighted images using a similar algorithm as above and was similarly expressed as the percentage of LV myocardial volume affected. The MSI was computed as follows: MSI = (AAR—infarct size) / AAR × 100 [13].

### Statistical analysis

Continuous variables were expressed as mean ± standard deviation or as median (25th percentile to 75th percentile) when variables lacked a normal distribution. An analysis of continuous variables was performed using the Student’s *t*-test and One-way ANOVA. Categorical variables were described as numbers (n) with percentages (%), and differences were analyzed by using Pearson χ^2^ or Fisher’s exact tests. Multivariate logistic regression analysis was performed using a step-wise backward selection process to determine the independent predictors of large myocardial infarcts (percent infarct volume ≥ mean of infarct size in the present study). Clinical variables (i.e., season, age, sex, diabetes mellitus, Killip class ≥3 on admission, LVEF ≤40%, leukocytosis, multivessel disease, and use of an angiotensin-converting enzyme [ACE] inhibitor or angiotensin receptor blocker [ARB] after primary PCI) were included in regression models. Criteria for the inclusion and exclusion of variables were set at 0.05 and 0.20, respectively. All tests were two-tailed, and *P* <0.05 was considered to be statistically significant. Statistical analyses were performed using the SAS software (version 9.2; SAS Institute Inc, Cary, NC, USA).

## Results

### Baseline characteristics

279 study patients were stratified into the Winter group (n = 56; 20.1%), the Spring group (n = 80; 28.7%), the Summer group (n = 76; 27.2%), and the Autumn group (n = 67; 24.0%) according to their STEMI occurrence season. Peak CK-MB levels tended to be lower in the Winter group than in other groups, but the difference was not significant (Table 1 and S1 Fig). In terms of concomitant medications used after revascularization, statins were administered less frequently in the Summer group than in the other groups (*P* = 0.018). Other demographic and clinical characteristics were not different among the four groups. Angiographic and procedural findings additionally showed no significant differences among the four groups (Table 1). The most common baseline TIMI flow grade was 0, and the baseline TIMI flows were not significantly different among the four groups. The most common infarct-related artery was the left anterior descending artery, and the prevalence of each involved vessel was similar among the four groups. The prevalence of multivessel disease and collateral flow were also similar among the four groups. The number of implanted stents, stent diameter, and stent length did not differ among the four groups. Angiographic no-reflow, final TIMI grade 3 after PCI, and final myocardial blush grade were not different among the four groups (Table 1).

**Table 1:** Baseline, angiographic and procedural characteristics.

	Overall population	Winter	Spring	Summer	Autumn	*P* value
	(n = 279)	(n = 56)	(n = 80)	(n = 76)	(n = 67)	
Age	59.2 ± 11.6	59.3 ± 10.5	60.3 ± 11.6	57.8 ± 11.9	59.2 ± 12.4	0.610
Male	225 (80.7)	45 (80.4)	59 (73.8)	65 (85.5)	56 (83.6)	0.265
Body mass index, *kg/m^2^*	24.8 ± 3.4	24.5 ± 3.8	25.0 ± 2.9	25.2 ± 3.9	24.5 ± 3.2	0.552
Current smoker	144 (58.2)	25 (44.6)	38 (47.5)	42 (55.3)	39 (58.2)	0.361
Hypertension	125 (44.8)	28 (50.0)	35 (43.8)	34 (44.7)	28 (41.8)	0.827
Diabetes mellitus	60 (21.5)	14 (25.0)	22 (27.5)	11 (14.5)	13 (19.4)	0.211
Dyslipidemia	50 (17.9)	12 (21.4)	13 (16.3)	11 (14.5)	14 (20.9)	0.651
Previous PCI	6 (2.2)	1 (1.8)	3 (3.8)	1 (1.3)	1 (1.5)	0.774
Previous CVA	10 (3.6)	3 (5.4)	1 (1.3)	3 (4.0)	3 (4.5)	0.540
*Times*, *minute*						
Symptom onset-to-balloon time	209.0 ± 136.8	219.8 ± 156.4	209.6 ± 134.0	195.9 ± 122.4	214.2 ± 140.1	0.768
Door-to-balloon time	61.9 ± 17.6	65.7 ± 16.5	59.7 ± 20.0	61.5 ± 16.1	61.9 ± 17.1	0.263
LVEF *(%)*	53.2 ± 10.8	53.2 ± 10.0	53.2 ± 10.3	53.6 ± 11.5	52.8 ± 11.3	0.977
*NT-proBNP, *pg/mL*	426.1 ± 105.8	498.0 ± 102.6	478.5 ± 120.1	336.5 ± 112.2	417.2 ± 181.8	0.840
Peak CK-MB, *ng/mL*	217.9 ± 162.1	179.4 ± 136.6	207.4 ± 181.2	228.7 ± 162.9	250.4 ± 151.6	0.088
*Concomitant medications*						
Aspirin	277 (99.3)	56 (100.0)	79 (98.8)	76 (100.0)	66 (98.5)	0.843
P2Y12 inhibitors	277 (99.3)	56 (100.0)	79 (98.8)	76 (100.0)	67 (100.0)	0.990
Statins	269 (96.4)	55 (98.2)	78 (97.5)	69 (90.8)	67 (100.0)	0.018
Beta blockers	255 (91.4)	48 (85.7)	73 (91.3)	71 (93.4)	63 (94.0)	0.349
ACE inhibitors/ARB	234 (83.9)	48 (85.7)	64 (80.0)	66 (86.8)	56 (83.6)	0.676
*Infarct-related artery*						0.917
Left anterior descending artery	138 (49.5)	26 (46.4)	41 (51.3)	38 (50.0)	33 (49.3)	
Left circumflex artery	29 (10.4)	7 (12.5)	8 (10.0)	7 (9.2)	7 (10.5)	
Right coronary artery	110 (39.4)	23 (41.1)	31 (38.8)	31 (40.8)	25 (37.3)	
Left main artery	2 (0.7)	0 (0.0)	0 (0.0)	0 (0.0)	2 (3.0)	
Multi-vessel disease	124 (44.4)	26 (46.4)	38 (47.5)	32 (42.1)	28 (41.8)	0.861
*TIMI flow grade before PCI*						0.344
0	215 (77.1)	45 (80.4)	60 (75.0)	56 (73.7)	54 (80.6)	
1	18 (6.5)	6 (10.7)	3 (3.8)	5 (6.6)	4 (6.0)	
2 or 3	46 (16.5)	5 (8.9)	17 (21.3)	15 (19.7)	9 (13.4)	
Presence of collateral flow	127 (51.6)	26 (55.3)	37 (52.9)	31 (43.7)	33 (56.9)	0.432
Final TIMI flow grade 3after PCI	264 (94.6)	53 (94.6)	77 (96.3)	73 (96.1)	61 (91.0)	0.711
Angiographic no reflow phenomenon	14 (5.0)	4 (7.1)	4 (5.0)	2 (2.6)	4 (6.0)	0.657
*Myocardial blush grade*						0.885
0	0 (0.0)	0 (0.0)	0 (0.0)	0 (0.0)	0 (0.0)	
1	2 (0.8)	0 (0.0)	0 (0.0)	1 (1.4)	1 (1.7)	
2	20 (8.1)	4 (8.5)	4 (5.6)	7 (9.7)	5 (8.6)	
3	226 (91.1)	43 (91.5)	67 (94.4)	64 (88.9)	52 (89.7)	
Aspiration thrombectomy	174 (62.4)	36 (64.3)	48 (60.0)	53 (69.7)	37 (55.2)	0.322
Use of GPIIb/IIIa inhibitor	43 (17.3)	10 (21.3)	9 (12.7)	12 (16.7)	12 (20.7)	0.560
PCI using stent	266 (95.3)	55 (98.2)	74 (92.5)	71 (93.4)	66 (98.5)	0.214
Number of implanted stent	1.2 ± 0.6	1.1 ± 0.4	1.3 ± 0.5	1.3 ± 0.6	1.3 ± 0.8	0.340
Stent diameter, *mm*	3.2 ± 0.6	3.2 ± 0.5	3.2 ± 0.5	3.2 ± 0.5	3.3 ± 0.7	0.788
Stent length, *mm*	30.1 ± 14.9	27.8 ± 13.0	30.3 ± 13.6	32.0 ± 16.2	29.7 ± 16.3	0.459

Data are presented as n (%) or mean ± standard deviation.

*Data of NT-proBNP were as available for 225 (80.6%) patients.

ACE = angiotensin-converting enzyme; ARB = angiotensin receptor blocker; BMI = body mass index; CK-MB = creatine kinase-myocardial band; CVA = cerebrovascular accident; GP = glycoprotein; LAD = left anterior descending artery; LCx = left circumflex artery; LVEF = left ventricular ejection fraction; MI = myocardial infarction; NT-proBNP = N-terminal prohormone of brain natriuretic peptide; PCI = percutaneous coronary intervention; RCA = right coronary artery; TIMI = thrombolysis in myocardial infarction.

### Analysis of CMR imaging findings

**Fig 2:**
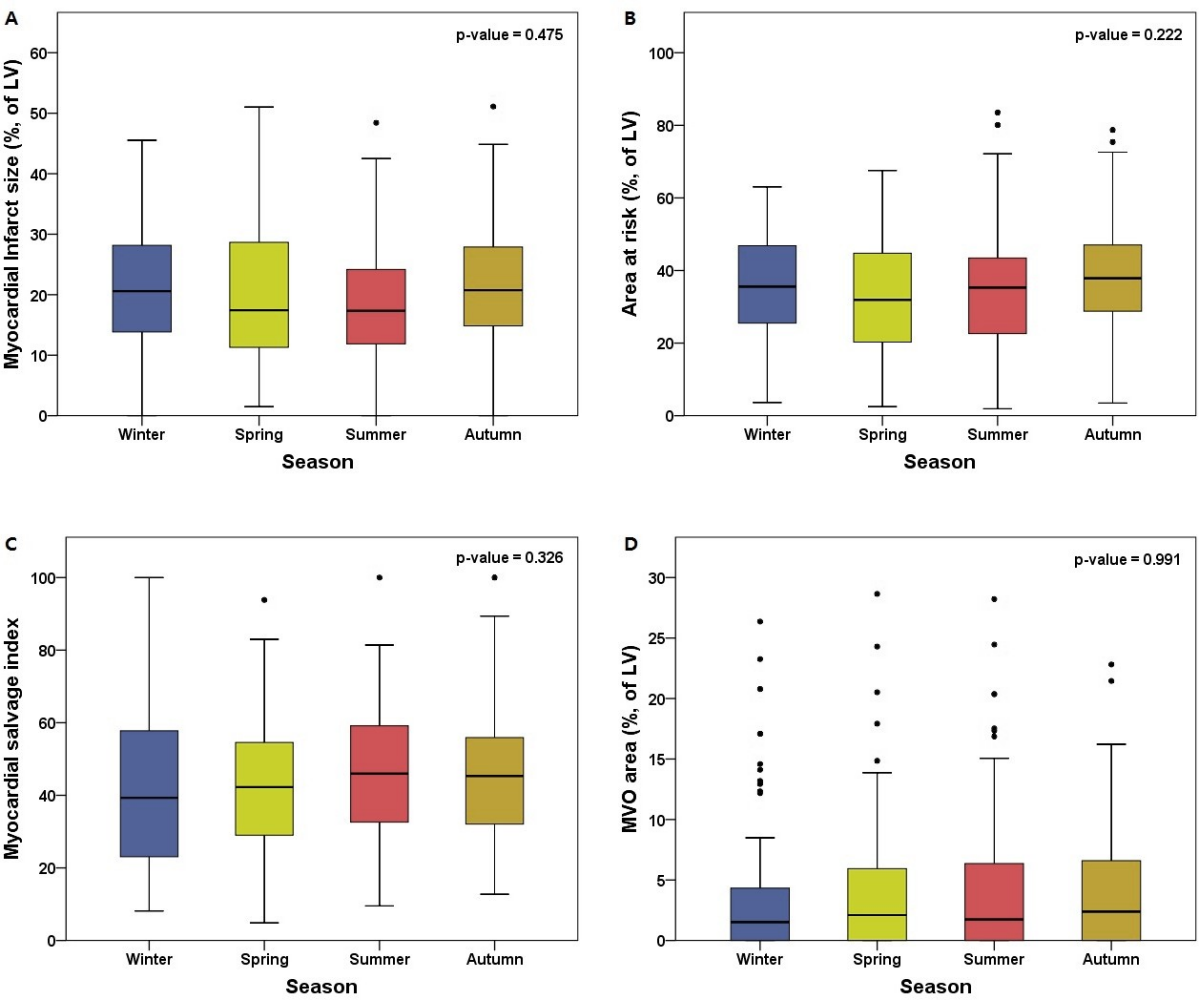
Cardiac magnetic resonance findings according to STEMI occurrence season. Boxplots show cardiac magnetic resonance data according to STEMI occurrence season and (A) myocardial infarct size, (B) extent of AAR, (C) MSI, and (D) MVO area. AAR = area at risk; LV = left ventricle; MSI = myocardial salvage index; MVO = microvascular obstruction.

CMR was performed for a median of 3.3 days (interquartile range: 2.8–4.2) after the index procedure, and intervals from primary PCI to CMR were not different among the four groups. The myocardial infarct size was not significantly different among the four groups (Winter group 21.0 ± 10.5% vs. Spring group 19.6 ± 11.5% vs. Summer group 18.6 ± 10.6% vs. Autumn group 21.1 ± 11.3%; *P* = 0.475). The extent of AAR (36.3 ± 13.8% vs. 32.4 ± 15.3% vs. 34.3 ± 16.2% vs. 37.4 ± 16.8%; *P* = 0.222), MVO area (4.4 ± 6.5% vs. 4.4 ± 6.0% vs. 4.6 ± 6.5% vs. 4.3 ± 5.3%; *P* = 0.991) and MSI (42.0 ± 22.5% vs. 41.6 ± 16.9% vs. 46.6 ± 18.8% vs. 45.2 ± 18.7%; *P* = 0.326) were similar among the four groups (Table 2 and Fig 2).

**Table 2:** Analysis of cardiac magnetic resonance findings.

	Overall population	Winter	Spring	Summer	Autumn	*P* value
	(n = 279)	(n = 56)	(n = 80)	(n = 76)	(n = 67)	
Myocardial infarct size *(%*, *of LV)*	20.0 ± 11.0	21.0 ± 10.5	19.6 ± 11.5	18.6 ± 10.6	21.1 ± 11.3	0.475
Area at risk *(%*, *of LV)*	34.9 ± 15.7	36.3 ± 13.8	32.4 ± 15.3	34.3 ± 16.2	37.4 ± 16.8	0.222
Myocardial salvage index	43.9 ± 19.1	42.0 ± 22.5	41.6 ± 16.9	46.6 ± 18.8	45.2 ± 18.7	0.326
MVO area *(%*, *of LV)*	4.4 ± 6.0	4.4 ± 6.5	4.4 ± 6.0	4.6 ± 6.5	4.3 ± 5.3	0.991
Hemorrhagic infarction	131 (47.0)	26 (46.4)	37 (46.3)	40 (52.6)	28 (41.8)	0.632
LV end diastolic volume *(mL)*	144.7 ± 35.1	146.8 ± 31.3	142.0 ± 32.4	150.0 ± 44.4	140.0 ± 28.6	0.307
LV end systolic volume *(mL)*	69.5 ± 31.0	71.7 ± 26.8	67.1 ± 27.5	73.7 ± 42.0	66.0 ± 22.2	0.392
LV ejection fraction *(%)*	53.2 ± 10.1	52.3 ± 9.8	54.0 ± 10.1	52.9 ± 11.6	53.5 ± 8.6	0.763
LV stroke volume *(mL)*	75.2 ± 16.5	75.1 ± 15.7	74.9 ± 15.6	76.7 ± 18.6	74.2 ± 16.0	0.885
LV cardiac output *(L/min)*	5.1 ± 1.1	5.3 ± 1.1	5.1 ± 1.0	5.1 ± 1.2	5.1 ± 1.1	0.447

Data are presented as n (%) or mean ± standard deviation.

LV = left ventricle (-ular); MVO = microvascular obstruction.

### Predictors of large myocardial infarct

In multivariate logistic regression analysis, all seasons were not potential predictors of large myocardial infarct. Independent predictors of large myocardial infarct (percentage of infarct volume of LV ≥20%) included male gender (odds ratio [OR] 1.93; 95% confidence interval [CI] 1.04–3.57; *P* = 0.037) and LVEF ≤40% (OR 6.11; 95% CI 2.26–16.53; *P* <0.001). Separately, the use of ACE inhibitor or ARB after primary PCI represented a uniquely negative predictor of large myocardial infarct (OR 0.50; 95% CI 0.26–0.97; *P* = 0.040) (Table 3).

**Table 3:** Predictors of large myocardial infarct.

	Odds ratio	95% CI	*P* value
***Season***			
Winter	1.21	0.67–2.17	0.529
Spring	0.79	0.47–1.32	0.365
Summer	0.78	0.46–1.32	0.346
Autumn	1.46	0.84–2.54	0.177
Age ≥65 years	1.05	0.63–1.73	0.857
Male	1.93	1.04–3.57	0.037
Diabetes mellitus	1.56	0.88–2.77	0.132
LVEF ≤40%	5.93	2.51–14.01	<0.001
^1^Leukocytosis	1.52	0.94–2.45	0.086
Multi-vessel disease	1.56	0.97–2.50	0.069
Use of ACE inhibitor or ARB after primary PCI	0.50	0.26–0.97	0.040

Large myocardial infarct was defined as a percentage of infarct volume ≥20%.

^1^Leukocytosis was defined as a WBC ≥11.0x10^3^/μL.

ACE = angiotensin-converting enzyme; ARB = angiotensin receptor blocker; CI = confidence interval; LVEF = left ventricular ejection fraction; PCI = percutaneous coronary intervention; WBC = white blood cell count.

### Harsh/Mild climate and advanced myocardial injury

The Winter and Summer groups were combined into the Harsh climate group, which was compared with the Mild climate group (consisting of the Spring and Autumn groups) in order to analyze the relationship between excessive temperature and myocardial injury. Of the 279 study patients, 132 suffered from STEMI in the winter and summer (Harsh climate group, 47.3%), while 147 suffered from STEMI in the spring and autumn (Mild climate group, 52.7%). The Harsh climate group had no differences with respect to myocardial infarct size, extent of AAR, and MSI compared to the Mild climate group (Table 4).

**Table 4:** Analysis of cardiac magnetic resonance findings.

	Overall population	Mild climate*	Harsh climate†	*P* value
	(n = 279)	(n = 147)	(n = 132)	
Myocardial Infarct size *(%*, *of LV)*	20.0 ± 11.0	20.3 ± 11.4	19.6 ± 10.6	0.581
Area at risk *(%*, *of LV)*	34.9 ± 15.7	34.7 ± 1.3	35.1 ± 15.2	0.806
Myocardial salvage index	43.9 ± 19.1	43.2 ± 17.8	44.6 ± 20.5	0.542
MVO area *(%*, *of LV)*	4.4 ± 6.0	4.3 ± 5.6	4.5 ± 6.5	0.840
Hemorrhagic infarction	131 (47.0)	65 (44.2)	66 (50.0)	0.334

Data are presented as n (%) or median (interquartile range)

*Mild climate was defined as spring and autumn.

†Harsh climate was defined as winter and summer.

LV = left ventricle (-ular); MVO = microvascular obstruction.

## Discussion

We investigated the association between seasonal variation and myocardial injury using CMR imaging in STEMI patients treated with primary PCI. The main finding of our study was that there was no significant difference of myocardial infarct size between winter, spring, summer, and autumn; furthermore, the extent of AAR, MSI, and MVO area were similar among the four seasons. Between harsh climates (winter and summer season) and mild climates (spring and autumn season), the nature of myocardial injury as assessed by CMR was not significantly different either. To the best of our knowledge, this is the first study to evaluate seasonal variations associated with myocardial injury as determined by CMR imaging data in STEMI patients. Our findings corresponded well with those of earlier studies that established an absence of seasonal effect on myocardial infarct size in acute MI patients [14]. Therefore, the results of our study may provide the causality and pathological mechanisms for the absence of seasonal variation in myocardial injury.

Previous studies have reported that environmental temperature may play an important role in the pathogenesis of acute MI [15]. Autonomic responses to cold including an increase in cardiac workload due to an increased need for oxygen, elevated sympathetic tone, and higher vascular resistance therefore it could contribute to increased rates of adverse events. Hematological changes to cold, such as an increase in platelets, red blood cell count, blood viscosity, and fibrinogen may also affect the onset of acute MI [3,16]. Kloner et al. [5] reported that there was a significant difference in infarct size depending on season, especially with smaller infarcts notably observed in the summer. However, the study was conducted in the pre-angioplasty era and, moreover, they analyzed patients who were treated with thrombolysis or only conventional medical therapy for heterogeneous MI. Luca et al. [14] separately showed no significant association between season and enzymatic infarct size in STEMI patients treated with primary PCI; however, they did not elucidate the underlying mechanisms. CMR imaging can assess relevant prognostic pathophysiological consequences of myocardial ischemia and reperfusion after acute MI [17,18] and is uniquely positioned to comprehensively evaluate the morphological, functional, and microvascular sequelae of postinfarct myocardium [7]. Therefore, we evaluated the association of seasons with acute MI and the prognostic pathophysiology of myocardial injury using CMR imaging data in STEMI patients treated with optimal revascularization and found that the myocardial and microvascular damage was similar throughout all seasons.

In addition, we investigated the relationship between excessive ambient temperature and myocardial injury in our subanalysis. Myocardial infarct size, AAR, MSI, and MVO as assessed by CMR imaging were not significantly different between the harsh climates (winter and summer season) and the mild climates (spring and autumn season). Bhaskaran et al. [15] reported that both hot and cold weather had adverse effects on short-term risk in patients with acute MI and in particular observed the stronger association of heat with STEMI versus other acute MIs. Keatinge et al. [19,20] reported that heat or cold stress might increase platelet and red blood cell counts, blood viscosity, and plasma cholesterol level. Specifically, heat or cold stress could activate the sympathetic nervous system and increase heart rates, thus exposure to a higher or lower temperature could contribute to increased incidences of acute MI [21,22]. Our study also investigated the relationship between higher or lower temperature and greater myocardial damage in patients with STEMI, but intense cold or heat did not result in advanced myocardial injury. A timely and optimal strategy of revascularization rather than seasonal effect may have a greater impact on myocardial injury in STEMI patients. Moreover, multivariate analysis showed that each season did not affect myocardial infarct size and that seasonal variation was not associated with advanced myocardial injury; instead, the use of an ACE inhibitor or ARB after primary PCI predicted lower incidences of large myocardial infarct. It is already well known that the use of ACE inhibitors or ARBs prevents advanced LV dysfunction and LV remodeling [23]. Our study analyzes the potential impact of season, underlying the importance of proper management independently from the weather, and contributes to a better understanding of the potential influence of environmental factors on STEMI patient management and prognosis.

### Study limitations

This study had several limitations. First, its design was nonrandomized, retrospective, and observational, which may have significantly affected the results attributed to confounding factors. Second, patients undergoing CMR may have been clinically stable with modest myocardial injury. Because only patients available for CMR were included, the sample size of our study was small and it may have limited our results. Third, due to the retrospective nature of our registry, we could not thoroughly identify detailed meteorological data or angiographic and clinical variables relating to the SYNTAX score in all study patients during follow-up. Fourth, South Korea has four distinct seasons, but this may represent a bias because the definition of the four seasons may be different in other geographic regions or countries. Finally, demographic population factors such as socioeconomic status and living area were not considered, and demographic factors could be one of the important factors affecting temperature-associated acute MI and its clinical outcomes, particularly in hot or cold weather.

## Conclusions

Seasonal effects could influence the incidence of acute MI, especially in the winter or summer; however, these are not associated with a larger extent of myocardial edema, less myocardial salvage, or greater myocardial infarct size as assessed by CMR imaging. Based on our study, seasonal variation may not affect advanced myocardial injury in STEMI patients undergoing primary PCI. Further investigation with potential therapeutic implications of these findings should be considered.

## Supporting information

S1 Fig Peak CK-MB levels according to STEMI occurrence date. Scatter plot shows peak CK-MB levels according to STEMI occurrence date.
CK-MB = creatine kinase myocardial band.
(TIF)

S1 Table Analysis of cardiac magnetic resonance findings. (DOC)

## Abbreviations

AAR: area at risk
MI: myocardial infarction
CMR: cardiovascular magnetic resonance
LV: left ventricle or–cular
MSI: myocardial salvage index
MVO: microvascular obstruction
PCI: percutaneous coronary intervention
STEMI: ST-segment elevation myocardial infarction
TIMI: thrombolysis in myocardial infarction
