# Supplementary figures and images for: Season and myocardial injury in patients with ST-segment elevation myocardial infarction: A cardiac magnetic resonance imaging study
Source: PLoS One. 2019 Feb 8;14(2):e0211807. doi: 10.1371/journal.pone.0211807 (PMC6368377; doi:10.1371/journal.pone.0211807)

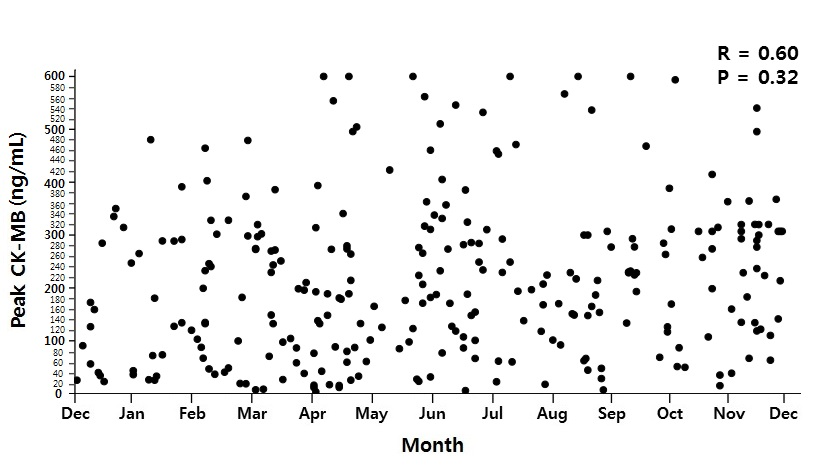

Supplement: S1 Fig — Scatter plot shows peak CK-MB levels according to STEMI occurrence date. CK-MB = creatine kinase myocardial band. (TIF) [file pone.0211807.s001.tif]
