# Supplementary material for: Season and myocardial injury in patients with ST-segment elevation myocardial infarction: A cardiac magnetic resonance imaging study
Source: PLoS One. 2019 Feb 8;14(2):e0211807. doi: 10.1371/journal.pone.0211807 (PMC6368377; doi:10.1371/journal.pone.0211807)
Supplement: S1 Table — (DOC) [file pone.0211807.s002.doc]

**S1 Table. Analysis of Cardiac Magnetic Resonance Findings**

|  | **Overall population**  **(n = 279)** | **Autumn + Winter**  **(n= 123)** | **Spring + Summer**  **(n = 156)** | ***P* value** |
| --- | --- | --- | --- | --- |
| Myocardial infarct size *(%, of LV)* | 20.0 ± 11.0 | 21.0 ± 10.9 | 19.1 ± 11.1 | 0.144 |
| Area at risk *(%, of LV)* | 34.9 ± 15.7 | 36.9 ± 15.4 | 33.3 ± 15.7 | 0.076 |
| Myocardial salvage index | 43.9 ± 19.1 | 43.7 ± 20.5 | 44.0 ± 17.9 | 0.899 |
| MVO area *(%, of LV)* | 4.4 ± 6.0 | 4.3 ± 5.8 | 4.5 ± 6.2 | 0.797 |
| Hemorrhagic infarction | 131 (47.0) | 54 (43.9) | 77 (49.4) | 0.365 |

Values are expressed as mean ± standard deviation or n (%).

LV = left ventricle (-ular); MVO = microvascular obstruction.
